# A Case of Familial Paroxysmal Non-kinesigenic Dyskinesia in Mainland China: A Clinical and Genetic Investigation

**DOI:** 10.7759/cureus.101172

**Published:** 2026-01-09

**Authors:** Wenhan Zhang, Jingwen Li, Ruichuan Xie, Zhongjiao Lu, Nan Zhao, Kan Wang, Yansheng Li, Shuang Li, Gang Wang, Li Gao

**Affiliations:** 1 Department of Neurology, Renji Hospital, School of Medicine, Shanghai Jiaotong University, Shanghai, CHN; 2 Department of Neurology, Huashan Hospital, Shanghai Medical College, National Center for Neurological Disorders, Fudan University, Shanghai, CHN; 3 Department of Neurology, Sanya People's Hospital, Sanya, CHN

**Keywords:** case report, mutation, myofibrillogenesis regulator, paroxysmal dyskinesias, paroxysmal non-kinesigenic dyskinesia

## Abstract

Paroxysmal non-kinesigenic dyskinesia (PNKD) is an autosomal dominant condition characterized by recurring dystonia or mixed chorea and ballism caused by emotional stress, exhaustion, coffee, alcohol, menstruation, and other factors. Here, we provide a large family PNKD pedigree from mainland China, where the same c.20C4T (p.Ala7Val) mutation in exon 1 of the PNKD gene was genetically confirmed to be present in nine affected individuals. Here, we report a rarely described Asian PNKD pedigree and aim to widen the clinical and genetic spectrum of PNKD while also providing fresh diagnostic insights for this rare condition.

## Introduction

Paroxysmal dyskinesias (PxDs) are a group of movement disorders characterized by recurrent episodes of involuntary movements without loss of consciousness. They are commonly classified into four subtypes, namely, paroxysmal kinesigenic dyskinesia (PKD), paroxysmal non-kinesigenic dyskinesia (PNKD), paroxysmal exertion-induced dyskinesia (PED), and paroxysmal hypnogenic dyskinesia (PHD), according to triggers, attack duration, frequency, and response to pharmacologic therapy. PNKD is the second most frequent subtype after PKD, with an estimated incidence of approximately one in 1,000,000. It typically presents with unilateral or bilateral involuntary movements, most often precipitated by alcohol or caffeine-containing beverages (e.g., coffee or tea). During episodes, patients commonly display dystonia, sometimes accompanied by chorea and/or ballism. Familial cases generally follow an autosomal dominant inheritance pattern with incomplete, age-dependent penetrance [1]. Although affected individuals share core clinical features, substantial inter-individual variability is observed. Recent genetic studies have identified pathogenic variants in PNKD1, also known as myofibrillogenesis regulator 1 (MR-1), a gene thought to be involved in cellular stress responses and regulation of neurotransmitter release, located on chromosome 2q35, as a major cause of familial disease. To date, three causative PNKD1 variants have been reported: p.Ala7Val, p.Ala9Val, and p.Ala33Pro.

Case reports of PNKD have predominantly originated from European populations, with only isolated familial reports from other regions. PNKD research in Asian populations is exceedingly restricted, with only single-family cases documented in Japan, Taiwan, mainland China, and elsewhere [1-4]. The present study reports a case and its four-generation pedigree, comprising a total of 37 affected individuals, with detailed clinical information collected from nine patients. Through the investigation of this family and a review of the relevant literature, we aim to broaden the understanding of the clinical spectrum, genetic background, and diagnostic considerations of PNKD in the Asian and also international populations.

## Case presentation

Patient information

**Figure 1:**
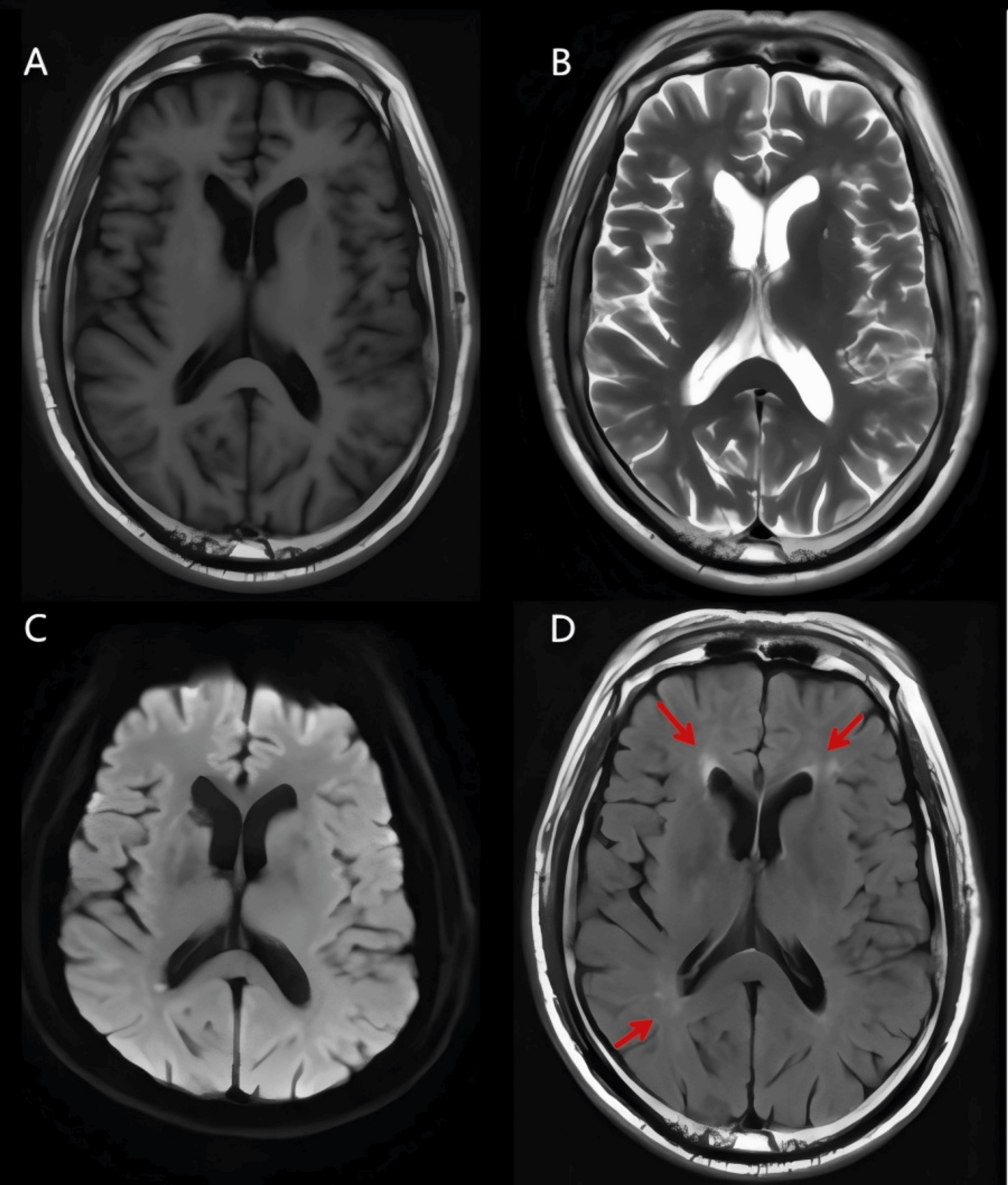
MRI findings of the patient Panels A-C showed no significant structural abnormalities on T1-weighted, T2-weighted, and DWI sequences, whereas panel D clearly demonstrated white matter lesions (marked by red arrows). MRI: magnetic resonance imaging; DWI: diffusion-weighted imaging

A 52-year-old man presented with an over 20-year history of recurrent involuntary limb movements. His initial episodes were triggered by alcohol intake or physical exertion, manifesting as spasms and stiffness of the left lower limb with foot extension and external rotation. Each attack lasted several minutes to half an hour, resolved spontaneously, and was not accompanied by loss of consciousness, incontinence, chest tightness, palpitations, or syncope. Anxiety, persistent discomfort in the left lower limb, and trouble walking were noted before attacks. Over the past year, attack frequency increased. Previous treatment with carbamazepine was ineffective. Past medical history and results of laboratory test were unremarkable. The cerebrospinal fluid (CSF) testing revealed no abnormalities. White matter lesions were identified by brain magnetic resonance imaging (MRI), but no other structural abnormalities were noted (Figure 1).

**Figure 2:**
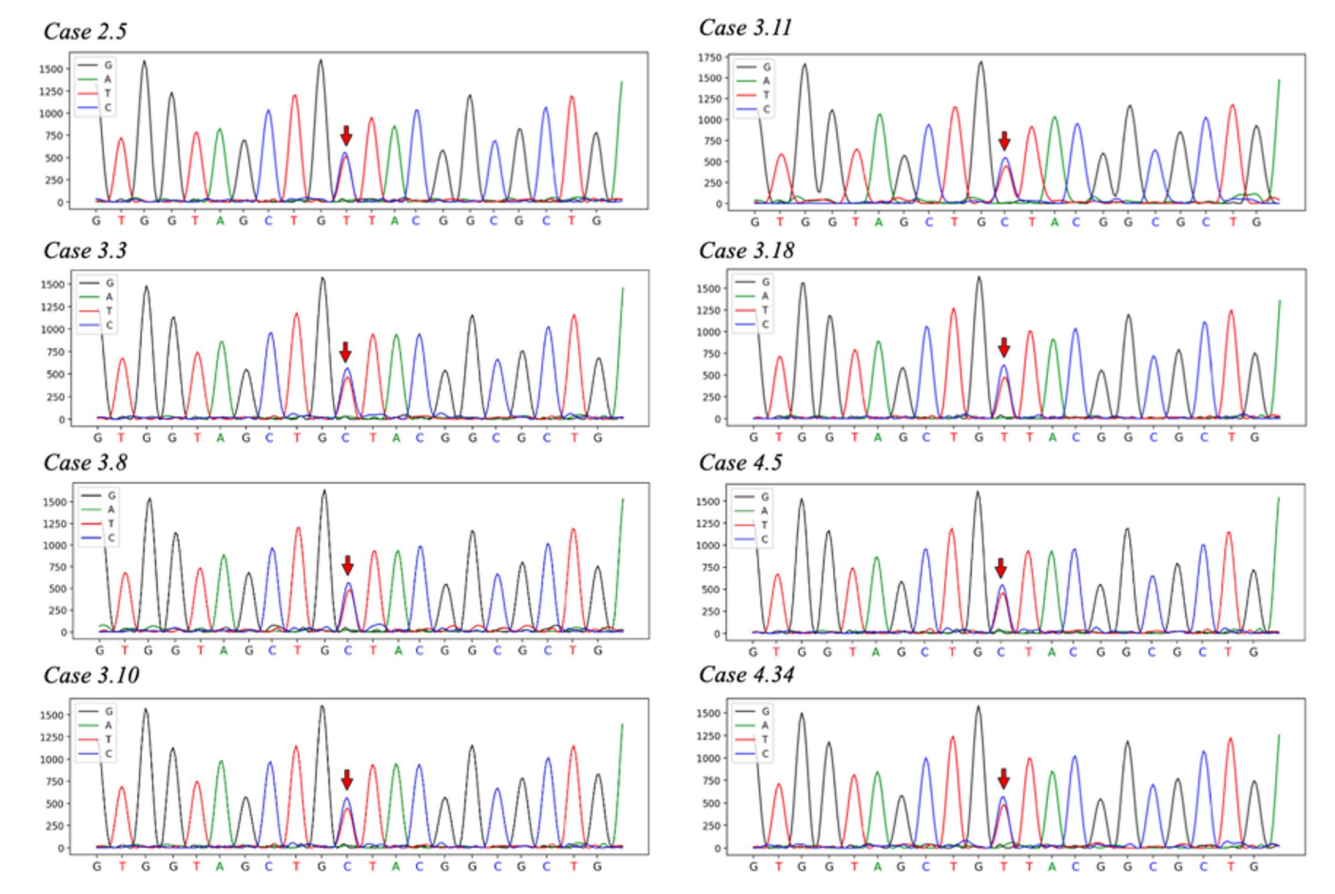
All of the documented patients showing c.20C4T (p.Ala7Val) mutation (red arrow) in PNKD/MR-1 gene exon 1

To validate the diagnosis of PNKD, targeted sequencing revealed a heterozygous c.20C>T (p.Ala7Val) mutation in exon 1 of the PNKD gene (Figure 2).

Pharmacologic treatment involved taking 1 mg of clonazepam twice a day, which produced a significant improvement. Later, the patient was given a thorough treatment plan that included non-pharmacologic treatments aiming at avoiding recognized PNKD triggers and changing lifestyles like getting enough sleep, reducing psychological stress, and sticking to a daily schedule.

Pedigree analysis of the family

**Figure 3:**
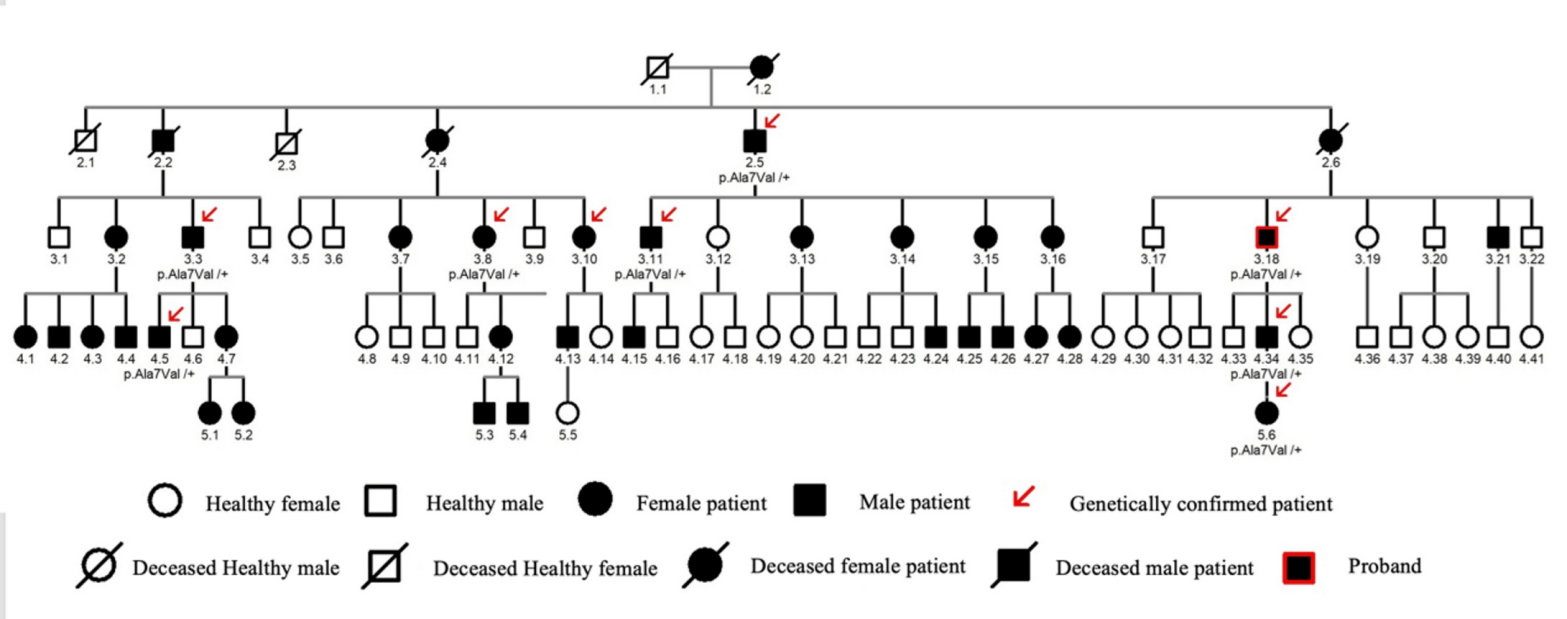
A pedigree chart depicting the familial relationships among all reported patients

Genetic testing and clinical data were collected from the proband's extended family because PNKD frequently manifested in familial instances. An autosomal dominant inheritance pattern was found in this four-generation Chinese mainland family with 37 afflicted members and nine documented patients, based on pedigree data and disease occurrence (Figure 3).

The age range of onset was 1-32 years old. Patients with PNKD exhibited distal limb rigidity, impaired activities of daily living (ADL), and involuntary body dystonia. Exposure to cold temperature, emotional stress, insufficient sleep, hunger, and exertion or fatigue were common triggers. Most patients had pharmacological treatment, with some responding to clonazepam. A summary of the comprehensive clinical data was provided in Table 1.

**Table 1:** Clinical characteristics of the nine documented patients with PNKD mutations PNKD: paroxysmal non-kinesigenic dyskinesia; y/o: years old; m/o: months old; NA: information not available; A: alcohol; ES: emotional stress; CT: cold temperature; H: hunger; EF: exertion or fatigue; IS: insufficient sleep; T: tea; V: vaccination; UST: unable to stand; FEA: facial expression alteration; SD: dysarthria; DT: dystonia; LOADL: loss of activities of daily living; IE: immobility of the extremities; LC: limb clonus; I: incontinence; BV: blurred vision

Patients	2.5	3.3	3.8	3.10	3.11	3.18	4.5	4.34	5.6
Gender	Male	Male	Female	Female	Male	Male	Male	Male	Female
Age	87 y/o	74 y/o	62 y/o	58 y/o	64 y/o	53 y/o	48 y/o	34 y/o	1 y/o
Age of onset	10 y/o	Childhood	Childhood	1 y/o	1 y/o	32 y/o	Childhood	Childhood	7 m/o
Frequency									
Earlier time	2-3 per month	1 per day	1 per day	1 per 3 days	1 per 20 days	N/A	Daily	N/A	N/A
Latest 2 years	Absent	1 per day	1 per 3 days	1 per 15 days	1 per 3 months	N/A	Monthly	N/A	N/A
Provoked ictal	10/10	8/10	4/10	2/10	2/10	7/10	7/10	7/10	N/A
Duration									
Average	3 min	N/A	30 min	30 min	30 min	30 min	N/A	N/A	N/A
Max	5 min	3 h	2 h	1 h	1 h	30 min	4 h	N/A	N/A
Min	3 s	1 h	30 min	30 min	30 min	mins	30 min	N/A	N/A
Trigger/precipitants	A, CT, ES, H	A, CT, IS, ES, H, EF	CT, IS, ES, H, EF	CT, ES, H, EF	CT, T, H, EF	A, EF	CT, IS, ES, H, EF	CT, T, ES, H	V
Attack manifestations	Asymptomatic	UST, FEA, SD, DT, LOADL, IE, LC (continuous tonic pattern)	UST, SD, DT, LOADL, IE	UST, SD, DT, LOADL, IE	UST, SD, DT, LOADL	D, LOADL, IE	BV, UST, FEA, SD, DT, LOADL, IE, LC (irregular twitching)	BV, UST, FEA, SD, DT, LOADL, IE, LC (irregular twitching)	LC (continuous tonic pattern)
Treatment	No	Clonazepam	Clonazepam	Clonazepam	N/A	Clonazepam	Clonazepam	Clonazepam	N/A
Efficacy	N/A	Asymptomatic	Effective	Effective	N/A	Effective	N/A	Effective	N/A

Case 2.5

The patient was an 87-year-old man with an onset at age 10. Attacks were triggered by alcohol, cold temperature, starvation, and emotional stress. In the early stage of the disease, attacks presented as somatic dystonia, speech disturbance, inability to stand, and loss of ADL. Attack frequency under identified triggers was 10/10. Initial attack frequency was 2-3 times per month; in the recent two years, attacks were resolved, and the patient remained asymptomatic.

Case 3.3

The patient was a 74-year-old man with a childhood onset. Precipitating factors included alcohol, cold temperature, insufficient sleep, starvation, and exertion or fatigue. Attack frequency under identified triggers was 8/10. Attacks phenomenology was characterized by body dystonia, facial expression alteration, speech disturbance, loss of ADL, occasional incontinence, and clonus of extremities presenting as continuous tonic movements. The attack frequency was once each day, and the duration ranged from one to three hours. Under the treatment of clonazepam, the patient is currently asymptomatic.

*Case 3.8* 

The patient was a 62-year-old woman with a childhood onset. Triggers included cold temperature, insufficient sleep, emotional stress, starvation, and exertion or fatigue. Clinical manifestations during attacks included inability to stand, speech disturbance, body dystonia, loss of ADL, and immobility of extremities. Attack frequency under identified triggers was 4/10. Initial attack frequency was once daily, later decreasing to once every three days. Duration ranged from 30 minutes to two hours. The patient was prescribed with clonazepam, and the attack frequency reduced greatly.

Case 3.10

The patient was a 58-year-old woman with an onset at the age of one, with a medical history of epilepsy. Triggers included cold temperature, emotional stress, starvation, and exertion or fatigue. Attack frequency under identified triggers was only 2/10, with the rest unknown. Attacks presented with speech disturbance, inability to stand, body dystonia, and loss of ADL. Initial attack frequency was once every three days, later reducing to once every 15 days, with a duration of 30 minutes to one hour. The patient is currently on clonazepam treatment with a moderate effect.

Case 3.11

The patient was a 64-year-old man with an onset at the age of one, also with a history of epilepsy. Triggers included cold temperature, tea consumption, starvation, and exertion or fatigue. Attack frequency under identified triggers was also only 2/10, with the rest unknown. Attacks were characterized by body dystonia, speech disturbance, and loss of ADL. Frequency during childhood averaged once every 20 days, decreasing to once every three months in adulthood. Duration averaged 30 minutes, with a maximum of one hour.

Case 3.18 (Proband)

The patient was a 53-year-old man who presented with an onset at age 32. Attacks were commonly triggered by alcohol and exertion or fatigue. The patient experienced prodromes as persistent left lower limb discomfort, affected gait, and anxiety. Clinical manifestations during attacks included dystonia and stiffness of the left lower limb. Duration ranged from several minutes to 30 minutes, with spontaneous resolution. Attack frequency increased over the past year. Pharmacologic therapy consisted of clonazepam 1 mg orally twice daily, resulting in a moderate symptomatic response.

Case 4.5

The patient was a 48-year-old man with a childhood onset. Triggers included cold temperature, insufficient sleep, starvation, emotional stress, and exertion or fatigue. Attack frequency under identified triggers was 7/10. Attacks were characterized by body dystonia, loss of ADL, and immobility of the extremities. Early attack frequency was almost daily, decreasing to once per month over the past two years. Duration ranged from 30 minutes to four hours. Clonazepam treatment was effective in controlling the attacks.

Case 4.34

The patient was a 34-year-old man with an onset at the age of three. Trigger factors included cold temperature, tea consumption, emotional stress, and starvation. Clinical manifestations during attacks were blurred vision, inability to stand, facial expression alteration, speech disturbance, body dystonia, loss of ADL, and immobility of the extremities, accompanied by irregular twitching limbs. Attack frequency under identified triggers was 7/10. The patient is currently treated with clonazepam.

Case 5.6

The patient was a one-year-old female with first episode at seven months following vaccination, presenting with bilateral hand tremor and absent stare, lasting several minutes and resolving spontaneously. No significant limb rigidity or other symptoms were found. Electroencephalogram (EEG) and brain MRI were normal.

Genetic mutation analysis

Whole-exome sequencing and pedigree analysis were performed in the nine affected members of this family. Eight patients were found to carry a heterozygous missense mutation c.20C>T (p.Ala7Val) in exon 1. No other mutations, including p.Ala9Val or p.Ala33Pro, were identified. Results are shown in Figure 2.

## Discussion

PNKD is a rare disorder with a lower global prevalence. Because of its rarity and clinical overlapping features with other paroxysmal conditions, the diagnosis can be challenging. Here, we reported a case and its four-generation pedigree, containing a total of 37 affected individuals, of whom nine patients carrying the MR-1 (p.Ala7Val) mutation have further clinical characterization. A PNKD family with detailed clinical information and genetic confirmation is unusual in the Asian population, which makes it highly unique. Together with the genetic results, their clinical characteristics were further contrasted with those reported in other pedigrees worldwide.

The most distinctive features of familial PNKD are the precipitators of attacks. Only a minority experienced spontaneous or even movement-induced attacks. Our study showed that attacks were commonly triggered by cold exposure or fasting in two-thirds of patients with PNKD. In addition, epilepsy coexisted in two of the nine patients, which was seldom reported in previous studies. In a survey of 49 patients with PNKD carrying MR-1 gene mutations, 98% reported tea or alcohol intake as the main trigger [5]. In contrast, in our present pedigree, cold exposure (77.8%) was identified as the most common precipitating factor, despite being rarely documented in previous reports, in which temperature changes were predominantly described as heat [4-6]. Emotional stress (55.6%) was the third most common trigger, whereas tea and coffee, typically regarded as the main precipitants, were reported by fewer than one-third of affected individuals. Notably, hunger (77.8%) was equally common as cold exposure in this cohort, and 66.7% of patients reported exertion or fatigue as a precipitating factor, which showed low prevalence in other previous reports [5,7]. Interestingly, Bruno et al. reported that exercise- and hunger-induced attacks occurred in 68% and 14% of MR-1-negative patients, respectively, but only in 12% and 6% of MR-1-positive patients [5]. The distinctive phenotypic features from patients with different PNKD genotypes, together with information from our cohort, suggest that unidentified potential genetic or epigenetic-environmental factors can modulate the final output of phenotypes. Furthermore, among the nine patients, two reported that only 20% of their attacks were associated with identifiable triggers, while the remaining attacks occurred either spontaneously or without a clearly identifiable cause.

The age of onset of PNKD ranges from infancy to early adolescence, with familial cases typically presenting early. Furthermore, longer attack durations and primary PNKD appeared to have an earlier age at onset compared with patients experiencing shorter attacks or with secondary PNKD [8]. In our pedigree, most of the nine documented patients experienced symptom onset during childhood. The proband, however, presented in early adulthood, while his offspring appeared to develop symptoms at progressively earlier ages, with his granddaughter experiencing attacks within the first year of life. This intra-familial variability may suggest a pattern of genetic anticipation, as has been previously observed in two southern European families [9].

PNKD attacks are typically characterized by dystonia, often accompanied by chorea or ballism. Aura, however, is found before attacks, with prevalence varying from 60% to 80% between families. Reported aura includes abnormal sensation of focal extremity, anxiety, speech disturbance, and weakness [4,5,10]. The proband exhibited prodromal symptoms of left lower limb discomfort and anxiety before attacks, whereas such data were unavailable for other family members. Attacks frequently begin in the perioral region or unilateral limbs, gradually involving the bilateral limbs and face, but without loss of consciousness. Involvement of facial muscles may result in speech impairment in some patients [1]. The most common attack manifestations of this family included involuntary body dystonia (77.8%), loss of ADL (77.8%), speech disturbance (66.7%), and inability to stand (55.6%). Approximately half of them exhibited limb rigidity or clonic movements, presenting as continuous rhythmic, irregular jerking, or spasm-like episodes. The attack frequency and duration also vary among PNKD patients. The frequency of attacks ranges from once or twice per day to only once or twice per year, and the duration is generally shorter than that of PKD, typically lasting 10 minutes to one hour, with some up to four hours [8,11,12]. Current evidence suggests that attack frequency generally decreases with age but does not differ between sexes [5]. Patients in our pedigree experienced attacks with variable frequency and duration, ranging from once a day to once every 20 days and lasting from a few seconds up to four hours. Most of them have a decrease in attack frequency and duration with age. Notably, the patient in case 2.5 exhibited markedly shorter attacks, lasting only a few seconds to minutes, and had become asymptomatic over time. In contrast, the proband reported a recent increase in both attack frequency and severity. This pattern is reminiscent of that observed in a Polish pedigree, in which all the male patients showed age-related increases in attack frequency and severity [13]. In female patients, endocrine appeared to modulate disease pattern, as symptom severity has been reported to fluctuate across ovulation, menstruation, pregnancy, and post-menopause, implicating a potential role for estrogen in attack modulation [2,13]. Although relevant data were limited in our cohort, these findings contribute to the heterogeneity of the disease.

This pedigree carried the p.A7V mutation in exon 1 of the PNKD gene, which was identical to the mutation reported in previous pedigrees from Taiwan and mainland China [1,4]. The PNKD gene was first mapped to chromosome 2q in 1996 through linkage analysis in 28 affected individuals and subsequently identified at 2q35 in 2004, where it was found to encode at least three isoforms including MR-1L, MR-1S, and MR-1M [14,15]. Three pathogenic mutations have been identified in PNKD to date including c.20C>T (p.Ala7Val) and c.26C>T (p.Ala9Val) in exon 1 and c.97G>C (p.Ala33Pro) in exon 2. Beyond MR-1, additional pathogenic genes for PNKD, including PNKD2, PRRT2, KCNMA1, and SLC2A1, have been identified. While mutations in these genes often involve ion channels or transporters, the molecular function of MR-1 mutations and the PNKD protein remains unknown. Mitochondrial dysfunction, impaired stress-response signaling, and dysregulated dopamine transmission are considered to be the key contributors [15-17]. 

Given the diversity of PNKD genotypes and the heterogeneity of clinical phenotypes, correlations between genotype and phenotype have been increasingly recognized. PNKD patients harboring different pathogenic gene mutations exhibit variability in clinical features and comorbidities. A study summarizing 19 families with MR-1 mutations reported that p.Ala7Val was the most frequently observed, followed by p.Ala9Val, with a single patient harboring p.Ala33Pro [4]. Interestingly, most asymptomatic carriers were females with a p.Ala9Val mutation. According to the analysis of multiple pedigrees from Bruno et al., PNKD patients carrying MR-1 mutations exhibited greater clinical homogeneity compared with those without MR-1 mutations, along with distinctive features including earlier age at onset, a higher likelihood of attacks precipitated by alcohol and caffeine, better responsiveness to benzodiazepines, and a presentation more consistent with the classical PNKD phenotype [5]. Furthermore, it is found that patients with MR-1 mutations are more frequently associated with migraine but better sleep benefit, whereas patients with KCNMA1 mutations are more often linked with epilepsy [2,5,16]. Notably, two patients in this pedigree had a history of epilepsy, a feature not previously reported in other PNKD cases. In addition, these two individuals experienced only two of the 10 provoked attacks as mentioned above. Dating back, a pedigree harboring a KCNMA1 mutation was reported in 2005 to have autosomal dominant generalized epilepsy combined with PxDs, in which the PxDs were alcohol-induced and described as KCNMA1-related PNKD-like attacks [18]. However, current literature generally considers PNKD associated with MR-1 mutations to be free of epilepsy, and to date, there have been no reports of PNKD coexisting with epilepsy [16]. All nine genetically tested patients in this pedigree were confirmed to carry MR-1 mutations. The presence of epilepsy in two of these patients is noteworthy and requires further follow-up to clarify the possible correlation between PNKD and epilepsy.

Most patients received clonazepam in this pedigree, which proved effective in alleviating disease severity including the proband, who had previously been treated with camazepam without improvement. Consistent with previous reports, PNKD patients generally respond well to benzodiazepines, whereas antiepileptic drugs effective for PKD show limited efficacy in PNKD.

## Conclusions

Overall, the clinical features observed in this pedigree were largely consistent with previous reports of familial PNKD, while the distinctive characteristics identified herein further enrich the clinical spectrum of the disorder and provide additional insights into its population heterogeneity. This pedigree also demonstrates that considerable variability exists among PNKD patients and that genetic factors likely account for only part of this variability. As a relatively typical PNKD pedigree, which remains rarely reported in mainland China, detailed clinical history and genetic findings of this family offer valuable diagnostic and clinical insights for Asian and global PNKD populations and lay the groundwork for future investigations into disease variability and potential modifiers as well.
